# The *Drosophila KIF1A* Homolog *unc-104* Is Important for Site-Specific Synapse Maturation

**DOI:** 10.3389/fncel.2016.00207

**Published:** 2016-09-05

**Authors:** Yao V. Zhang, Shabab B. Hannan, Zeenna A. Stapper, Jeannine V. Kern, Thomas R. Jahn, Tobias M. Rasse

**Affiliations:** ^1^Junior Research Group Synaptic Plasticity, Hertie-Institute for Clinical Brain Research, University of TübingenTübingen, Germany; ^2^Graudate School of Cellular and Molecular Neuroscience, University of TübingenTübingen, Germany; ^3^The Picower Institute for Learning and Memory, Department of Biology and Brain and Cognitive Sciences, Massachusetts Institute of TechnologyCambridge, MA, USA; ^4^Schaller Research Group at the University of Heidelberg and DKFZ, Proteostasis in Neurodegenerative Disease (B180), German Cancer Research CenterHeidelberg, Germany

**Keywords:** *KIF1A*, *unc-104*, kinesin-3, hereditary spastic paraplegia, *Drosophila*, synapse maturation, neuromuscular junction, Rab3

## Abstract

Mutations in the kinesin-3 family member *KIF1A* have been associated with hereditary spastic paraplegia (HSP), hereditary and sensory autonomic neuropathy type 2 (HSAN2) and non-syndromic intellectual disability (ID). Both autosomal recessive and autosomal dominant forms of inheritance have been reported. Loss of *KIF1A* or its homolog *unc-104* causes early postnatal or embryonic lethality in mice and *Drosophila*, respectively. In this study, we use a previously described hypomorphic allele of *unc-104*, *unc-104^bris^*, to investigate the impact of partial loss-of-function of kinesin-3 on synapse maturation at the *Drosophila* neuromuscular junction (NMJ). *Unc-104^bris^* mutants exhibit structural defects where a subset of synapses at the NMJ lack all investigated active zone (AZ) proteins, suggesting a complete failure in the formation of the cytomatrix at the active zone (CAZ) at these sites. Modulating synaptic Bruchpilot (Brp) levels by ectopic overexpression or RNAi-mediated knockdown suggests that the loss of AZ components such as Ca^2+^ channels and Liprin-α is caused by impaired kinesin-3 based transport rather than due to the absence of the key AZ organizer protein, Brp. In addition to defects in CAZ assembly, *unc-104^bris^* mutants display further defects such as depletion of dense core and synaptic vesicle (SV) markers from the NMJ. Notably, the level of Rab3, which is important for the allocation of AZ proteins to individual release sites, was severely reduced at *unc-104^bris^* mutant NMJs. Overexpression of Rab3 partially ameliorates synaptic phenotypes of *unc-104^bris^* larvae, suggesting that lack of presynaptic Rab3 contributes to defects in synapse maturation.

## Introduction

The active zone (AZ) is a specialized presynaptic site for Ca^2+^ mediated synaptic vesicle (SV) fusion and neurotransmitter release. Changes in the efficacy of the presynaptic release machinery at the AZ are crucial for synaptic plasticity during learning and memory and are implicated in several neurodegenerative diseases. The fruit fly neuromuscular junction (NMJ) has been widely used as a model to study synaptic transmission in health and disease due to its accessibility and an increasing array of genetic tools available in *Drosophila* (Südhof, 2012; Frank et al., 2013). Recent studies have resulted in remarkable progress in identifying and characterizing the proteins constituting the presynaptic AZ at the *Drosophila* NMJ. These include: cytomatrix at the active zone (CAZ)-associated structural protein (CAST)/ELKS-1 homolog Bruchpilot (Brp; Kittel et al., 2006; Wagh et al., 2006), voltage gated Ca^2+^ channel subunit Cacophony (Cac; Kawasaki et al., 2004), Syd-2/Liprin-α (Zhen and Jin, 1999; Kaufmann et al., 2002), Serine-arginine protein kinase at 79D (SRPK79D; Johnson et al., 2009; Nieratschker et al., 2009), RhoGAP-like protein DSyd-1 (Owald et al., 2010), Fife (Bruckner et al., 2012), and RIM binding protein (Liu et al., 2011). AZ proteins are involved in maintaining various aspects of the structural integrity and function of synapses (Ackermann et al., 2015).

Synaptogenesis requires orchestration of precisely timed sequential events. Here, we use the term core synaptic assembly to refer to the initial stages of the assembly of synaptic proteins following contact between a pre- and post-synaptic cell, leading to a rudimentary synapse. Following core synaptic assembly, synapse maturation ensues, leading to the recruitment of additional synaptic components and formation of highly specialized pre-and post-synaptic structures that result in a several fold increase in the efficacy and strength of nascent or previously silent synapses (Rasse et al., 2005; Fouquet et al., 2009; Owald and Sigrist, 2009; Owald et al., 2010). Nucleation of the CAZ is an early step in synapse maturation (Graf et al., 2009; Bae et al., 2016). An important role has been attributed to the small GTPase Rab3 in the distribution of AZ proteins to individual release sites at the *Drosophila* NMJ (Graf et al., 2009). Rab3 is a member of the Rab family of proteins implicated in SV exocytosis; Rab3 regulators and effectors have been identified, and include Rab3-GAP, Rab3-GEF, Rab-GDI, Rabphilin-3A, and RIM (Geppert and Sudhof, 1998; Richmond and Broadie, 2002). Members of the Rab3 family, including Rab3-GAP and Rab3-GEF, have also been implicated in synaptic homeostasis and presynaptic maturation (Giagtzoglou et al., 2009; Graf et al., 2009; Müller et al., 2011; Chen et al., 2015; Bae et al., 2016). In *rab3* mutants, Brp clusters aberrantly at a few AZs while most other synapses are completely devoid of Brp (Graf et al., 2009). Giant Brp positive (Brp^+^) puncta and negative (Brp^−^) synapses are intermingled within the same bouton suggesting that Rab3 might be important for the control of AZ nucleation and/or maturation in a site-specific manner (Graf et al., 2009). A similar distribution pattern of Brp^+^ and Brp^−^ synapses have been described for a hypomorphic allele of *unc-104* (Kern et al., 2013), the *Drosophila* homolog of *KIF1A* that encodes a kinesin-3 type motor protein, raising the possibility that common mechanisms might cause this defect.

Synapses often form at the tip of neuronal processes, which can be a long distance away from the cell body (CB). Hence, synaptic proteins, signaling molecules, and necessary organelles, need to be transported by motor proteins along cytoskeletal tracks to their destination to be integrated into the synaptic machinery, implying that synaptic formation is intimately linked to anterograde axonal transport (Bury and Sabo, 2015). As the major anterograde molecular motors, kinesins are responsible for the transport of various types of synaptic cargo. Mutations in *KIF1A* have been associated with the motoneuron disease hereditary spastic paraplegia subtype 30 (SPG30; Erlich et al., 2011; Klebe et al., 2012; Citterio et al., 2015; Lee et al., 2015; Ylikallio et al., 2015), hereditary and sensory autonomic neuropathy type 2 (HSAN2; Rivière et al., 2011), and non-syndromic intellectual disability (ID; Hamdan et al., 2011). Autosomal recessive (Rivière et al., 2011) and autosomal dominant forms of inheritance (Citterio et al., 2015; Ylikallio et al., 2015) as well as heterozygous *de novo* mutations (Hamdan et al., 2011; Esmaeeli Nieh et al., 2015; Lee et al., 2015; Ohba et al., 2015; Hotchkiss et al., 2016) have been implicated in *KIF1A*-associated human diseases, suggesting loss-of-function, toxic gain-of-function or dominant negative modes of impairment.

Homozygous *KIF1A*-knockout mice are significantly smaller than age-matched control littermates, show severe motor and sensory deficits and substantial neuronal death, and die shortly after birth (Yonekawa et al., 1998). In *Drosophila*, *unc-104* null mutant embryos are paralyzed and fail to hatch (Pack-Chung et al., 2007). Motoneuron growth cones of *unc-104* null embryos do not mature into synaptic boutons, very few AZs form, and SVs are rare at the NMJ (Pack-Chung et al., 2007). To model the effect of partial loss of *KIF1A* function, animal models with milder mutations such as hypomorphs are useful. Employing the hypomorphic allele of the *Drosophila* kinesin-3, *unc-104^bris^*, caused by a point mutation in the forkhead associated (FHA) domain, we demonstrated that reduced kinesin-3 motor function results in late larval lethality, locomotion defects, pre- and post-synaptic structural defects, accumulation of Brp at the CB region of the larval ventral nerve cord (VNC), increased NMJ length, increased synaptic bouton number, and reduced synaptic bouton size (Kern et al., 2013). Although a fraction of synapses lack Brp at *unc-104^bris^* NMJs, suggesting impaired AZ nucleation or maturation, the effect of *unc-104^bris^* on the regulation of these processes remains elusive. It is, for example, unclear how unc-104 affects the molecular composition of AZs including the localization of Cac, Liprin-α, and SRPK79D to Brp^+^ or Brp^−^ synapses and the localization of SV proteins in boutons. In this study, we demonstrate that AZ maturation is abolished in a subset of synapses at *unc-104^bris^* mutant NMJs. Major AZ proteins, including Brp, Cac, Liprin-α and SRPK79D, are absent from these synapses. The localization of SV proteins as well as dense core vesicles (DCVs) is severely impaired at *unc-104^bris^* mutant NMJs. We found that Rab3 protein levels are severely reduced at *unc-104^bris^* mutant NMJs and that ectopic expression of Rab3 was sufficient to ameliorate pre- and post-synaptic defects without altering the VNC transport phenotype in *unc-104^bris^*.

## Materials and Methods

### Molecular Biology

To construct Liprin-α-GFP, Liprin-α cDNA (LD 33094) was C-terminally tagged with GFP using standard cloning technologies. Neuronal expression was induced using a PP2A minimal promotor element (3R: 18,169,704‥.18,174,719; gift from Aaron DiAntonio) integrated into a modified pCaSpeR vector. An electronic vector map is available upon request.

### Fly Stocks

Flies were cultured as previously described (Butzlaff et al., 2015). w1118, elav(X)-Gal4, *unc-104*^d11024^ and UAS-Cac-GFP flies were obtained from Bloomington Stock Center. D42-Gal4 (GFP tagged atrial natriuretic factor (ANF-GFP), mito-GFP) recombination stocks were received as a generous gift from William Saxton. UAS-Brp-RNAi (C8) were obtained from Stephan Sigrist (Wagh et al., 2006). UAS-SRPK79D-GFP was obtained from Erich Buchner. UAS-Rab3 flies were obtained from Aaron DiAntonio.

### Immunohistochemistry

Larval dissections (mid-3rd instar stage) were performed essentially as previously described (Fuger et al., 2012; Butzlaff et al., 2015). In brief, larvae were dissected using fine dissection scissors in Ca^2+^-free HL3 solution and fixed in 4% paraformaldehyde in PBS either for 3 min (staining with native fluorescent proteins) or for 10 min (for staining with only immunofluorescent labeling). Overnight primary antibody incubation was performed at 4°C in PBS with 0.05% Triton-X and 5% normal goat serum as blocking solution. Fillets were then washed and incubated with fluorescent-conjugated secondary antibodies at room temperature for 2 h and then washed again. Finally, larval fillets were mounted on a glass slide in mounting media (Vectashield, Vector). Primary antibodies used were: mouse monoclonal anti-Brp (NC82) at 1:100 (Developmental Studies Hybidoma Bank), mouse monoclonal CSP (DCSP-2) at 1:50 (Developmental Studies Hybidoma Bank) rabbit anti-glutamate receptor subunit IIC (GluRIIC) at 1:2000 (Stephan Sigrist), rabbit anti-vesicular glutamate transporter (VGlut) at 1:1000 (Hermann Aberle) and rabbit anti-Rab3 at 1:1000. Fluorescent-conjugated secondary antibodies used were: goat anti-mouse Alexa 488 or Alexa 568 and goat anti-rabbit Alexa 488 or Alexa 568 (Molecular Probes) or goat anti-mouse Atto 647 (Sigma). Goat anti-horseradish peroxidase (HRP) conjugated with Cy3 (Dianova) was added together with secondary antibodies. All were used at a dilution of 1:500. Images were captured using a Zeiss LSM 710 or 780 confocal microscope, with a 40× plan Apochromat 1.3 N.A. oil objective and the ZEN software. ImageJ (NIH) was used for image processing and analysis.

### Sample Preparation for Electron Microscopy

4% PFA (in PBS) was used to fix larval fillets for 10 min at room temperature followed by overnight fixation in 2.5% glutaraldehyde (in PBS) at 4°C. For postfixation, samples were treated with 1% osmium tetroxide in 100 mM phosphate buffer, pH 7.2, on ice for 1 h. Next, fillets were rinsed with water, treated with 1% aqueous uranyl acetate (UA) for 1 h at 4°C, dehydrated through a graded series of ethanol concentrations prior to overnight storage in liquid Epon. Samples were pinned on a dissection pad and VNCs were dissected using sharp insect pins, embedded in Epon, and polymerized for 48 h at 60°C. Ultrathin sections were stained with UA and lead citrate and viewed in a FEI-Tecnai Spirt, 120 kV electron microscope equipped with a Gatan USC 4000 camera.

### Quantifications of SVs, Mitochondria and AZs in Electron Micrographs

File names of electron microscopic sections from larval VNC were annotated prior to their analysis in a double-blind manner. Multiple cells were counted from each image and the number of SVs, mitochondria and AZs in synaptic regions were counted and recorded. The number of SVs and mitochondria were normalized by the cell area while the number of AZs were normalized by the length of the membrane in the corresponding cells.

### Statistical Analysis

Statistical analysis was performed using the software PRISM 6. Sample errors are given as standard error of the mean (SEM). Data were first tested for normality prior to analysis either by student’s *t*-test (two groups) or by a one-way analysis of variance followed by a Tukey’s multiple comparison test. Non-normally distributed data were analyzed by using either a Mann-Whitney test (two groups) or a Kruskal-Wallis test for multiple groups. The following alpha levels were used for all tests: *p < 0.05; **p < 0.01; ***p < 0.001.

## Results

### Essential Components of the AZ are Absent at a Fraction of Synapses in *unc-104^bris^* Mutant NMJs

**Figure 1:**
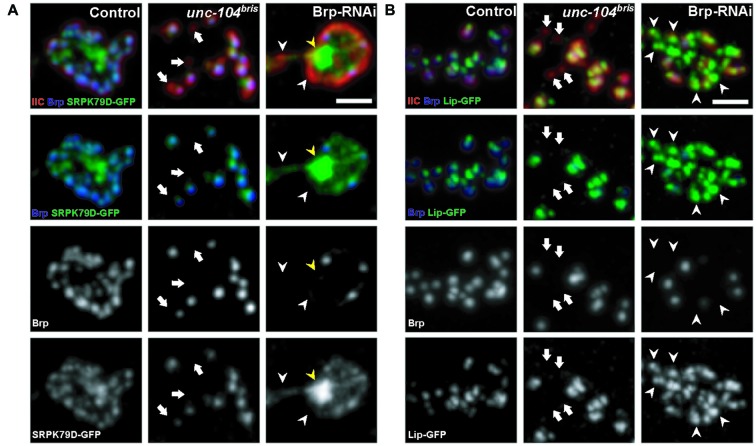
**Localization of presynaptic components at the neuromuscular junction (NMJ) of *unc-104^bris^* mutant and Bruchpilot (Brp) RNAi expressing larvae. (A,B)** Synaptic bouton at the NMJ costained with antibodies against postsynaptic glutamate receptor subunit IIC (GluRIIC, red in upper panel) and presynaptic Brp (blue in upper panel, gray in 3rd panel). Clustering pattern of GFP-tagged serine-arginine protein kinase at 79D (SRPK79D-GFP; **A**) and Liprin-α (Lip-GFP; **B**) respectively in synaptic bouton. **(A)** SRPK79D-GFP did not concentrate at Brp negative (Brp^−^) synapses in *unc-104^bris^* mutants (arrows). At the NMJ of larvae expressing BRP RNAi pan-neuronally (Brp-RNAi), SRPK79D-GFP failed to concentrate at Brp^−^ synapses (white arrowheads) and large aggregation of SRPK79D-GFP is evident in the bouton (yellow arrowheads). **(B)** Lip-GFP does not localize to Brp^−^ synapses in *unc-104^bris^* larvae (arrows), whereas in Brp-RNAi animals Lip-GFP is present at Brp^−^ synapses (white arrowheads). Genotypes in **(A)**: control (*elavX-Gal4/+*;;*UAS-Srpk79d-GFP/+*), *unc-104^bris^* (*elavX-Gal4/+*;*unc-104^bris^*/unc-104^d11024^;*UAS-Srpk79d-GFP/+*), Brp-RNAi (*elavX-Gal4/+*;;*UAS-Srpk79d-GFP/UAS-Brp-RNAi*). Genotypes in **(B)**: control (*elavX-Gal4/+*;;*PP2A-Lip-GFP/+*), *unc-104^bris^* (*elavX-Gal4/+*;unc*-104^bris^*/unc-104^d11024^;*PP2A-Lip-GFP/+*), Brp-RNAi (*elavX-Gal4/+*;;*PP2A-Lip-GFP/UAS-Brp-RNAi)*. Experiments were performed at 29°C. Scale bars: 2 μm.

**Figure 2:**
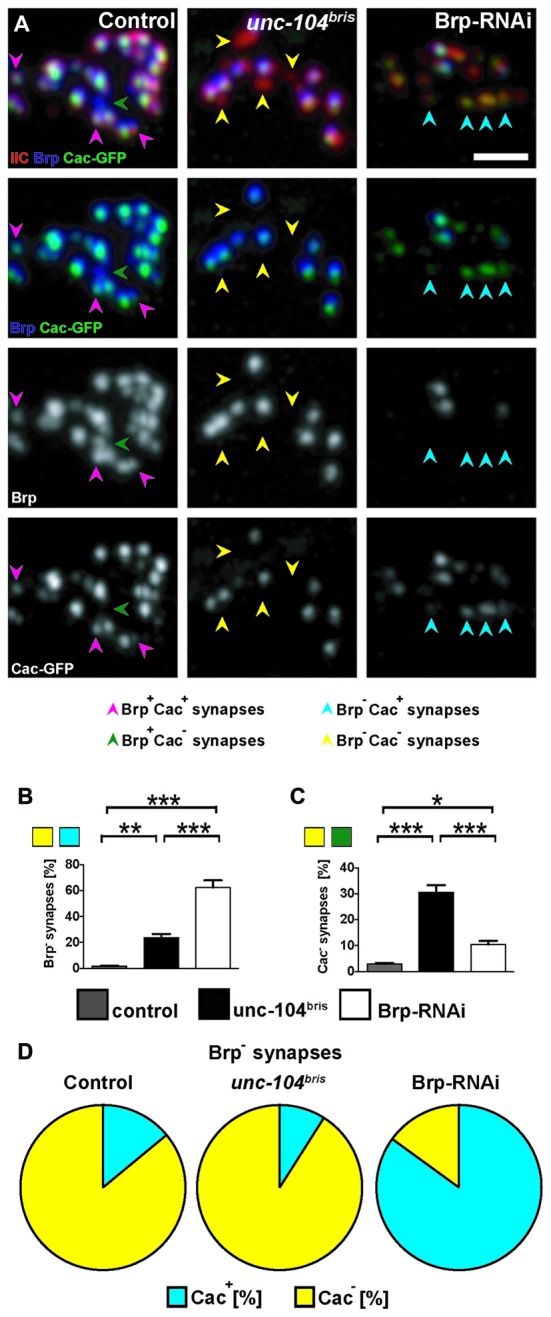
**Cacophony (Cac) at the NMJ of *unc-104^bris^* mutant and Brp-RNAi expressing larvae. (A–C)** Pattern of Cac clustering observed by expressing GFP-tagged Cac (Cac-GFP) at the active zones (AZs) in control, *unc-104^bris^* and Brp-RNAi larvae **(A)**. Brp^−^ synapses in *unc-104^bris^* animals are also devoid of Cac-GFP (yellow arrowheads). In contrast, Brp^−^ synapses in Brp-RNAi animals are mostly Cac-GFP positive (blue arrowheads). **(B,C)** Quantifications of the percentages of Brp^−^ and Cac-GFP negative (Cac^−^) synapses. **(D)** Pie-charts showing the proportion of Brp^−^ synapses that are Cac-GFP positive (Cac^+^) and Cac^−^. Genotypes in **(A)**: control (*elavX-Gal4/+*;;*UAS-Cac-GFP/+*), *unc-104^bris^* (*elavX-Gal4/+*;unc*-104^bris^*/unc-104^d11024^;*UAS-Cac-GFP/+*), Brp-RNAi (*elavX-Gal4/+*;;*UAS-Brp-RNAi/UAS-Cac-GFP*). Experiments were performed at 29°C. Scale bar: 2 μm. Statistical test: one-way ANOVA followed by Tukey’s Multiple Comparison Test. *p < 0.05; **p < 0.01; ***p < 0.001. Error bars represent SEM.

We have previously shown that ~25% of synapses in *unc-104^bris/-^* mutant NMJs (*unc-104^bris^* transheterozygote hereafter referred to as *unc-104^bris^*) lack the presynaptic AZ scaffold protein Brp (Kern et al., 2013). In order to gain more insight into the role of kinesin-3 in the delivery and incorporation of presynaptic components into the AZ, we expressed GFP-tagged AZ proteins SRPK79D, Liprin-α, and Cac, and examined their localization in *unc-104^bris^* mutant NMJs. Larvae were co-stained for pre- and post-synaptic markers, Brp and glutamate receptor subunit IIC (GluRIIC; Qin et al., 2005), respectively. At control NMJs, AZ components SRPK79D, Liprin-α, and Cac all colocalized with Brp (Figures 1A,B, 2A). At *unc-104^bris^* mutant NMJs, a vast majority of Brp^−^ synapses were also negative for SRPK79D, Liprin-α and Cac (Figures 1A,B, arrows; Figure 2A, yellow arrowheads) while Brp^+^ synapses successfully recruited SRPK79D, Liprin-α, and Cac (Figures 1A,B, 2A). Since a fraction of synapses in *unc-104^bris^* mutant NMJs did not contain any of the essential AZ components, we conclude that the CAZ failed to assemble in these synapses.

Brp has been shown to play an essential role for AZ maturation in *Drosophila* (Kittel et al., 2006). Given that other examined AZ components follow a pattern of localization similar to Brp, we asked if the failed AZ maturation at a fraction of synapses in *unc-104^bris^* mutant NMJs may be secondary to the reduced Brp level at the NMJ. We mimicked the reduced Brp abundance at the NMJs observed in *unc-104^bris^* mutant by knocking down Brp levels pan-neuronally in otherwise wildtype genetic background using a well characterized Brp-RNAi construct (Wagh et al., 2006).

Pan-neuronal Brp knockdown in control background resulted in a large fraction of synapses devoid of pre-synaptic Brp at AZs (Figures 1A,B white arrowheads; Figure 2A, blue arrowheads). In Brp-RNAi expressing larvae, SRPK79D-GFP signal failed to properly localize to Brp^−^ synapses (Figure 1A, white arrowheads). Rather, it was diffusely distributed throughout the bouton, and large, bright SRPK79D-GFP puncta were frequently observed in the center of synaptic boutons (Figure 1A, yellow arrowheads), possibly a result of SRPK79D self-aggregation due to the reduced number of AZs. These results suggest that the SRPK79D association with Brp is instructive for its AZ localization. Brp knockdown in the wildtype background did not, however, alter the gross localization of Liprin-α (Figure 1B), and unlike in *unc-104^bris^* mutants, the large percentage of Brp^−^ synapses in Brp-RNAi larvae are Liprin-α positive (Liprin-α^+^; Figure 1B, arrowheads).

In *unc-104^bris^* larvae, Brp^−^ synapses are generally also Cac negative (Cac^−^; Figure 2A, yellow arrowheads; Figure 2D). However, NMJs with reduced Brp due to pan-neuronal expression of Brp-RNAi in the wildtype background does not exhibit the same pattern of Cac mis-localization; most of the Brp^−^ synapses are Cac positive (Cac^+^; Figure 2A, blue arrowheads; Figure 2D). Brp-knockdown in the wildtype background dramatically increased the fraction of Brp^−^ synapses (~50% in Brp-RNAi vs. ~2% in control, *P* < 0.001; Figure 2B), a significant fraction of which are Cac^+^ (Figure 2D). Although the number of Cac^−^ synapses increased sharply in *unc-104^bris^* mutants (~30% in *unc-104^bris^* vs. ~3% in control, *P* < 0.001; Figure 2C), in Brp-RNAi animals Cac^−^ synapses increased only mildly when compared to the control (~10% in Brp-RNAi vs. ~3% in control, *P* < 0.05; Figure 2C). Taken together, these results suggest that the failed localization of core AZs components to a subset of AZs in *unc-104^bris^* mutants is unlikely secondary to reduced Brp at the NMJ.

### Brp Knockdown or Overexpression Modifies the AZ Maturation Phenotype at *unc-104^bris^* Mutants

**Figure 3:**
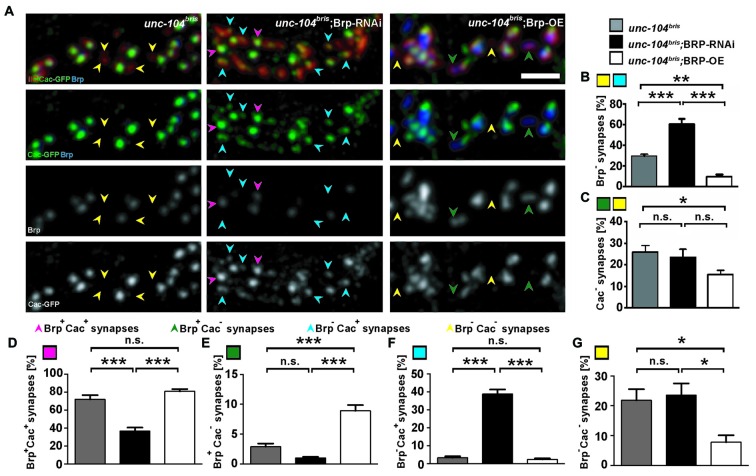
**Impact of varying Brp levels on presynaptic Brp and Cac in *unc-104*^bris^ background. (A)** A stretch of synaptic boutons at the NMJ costained with antibodies against GluRIIC (red, upper panel) and Brp (blue in upper panel, gray in 3rd panel). Cac was visualized by expression of Cac-GFP (green in 2nd panel, gray in lower panel). Mutant *unc-104^bris^* NMJs are characterized by frequent absence of both Brp and Cac-GFP (yellow arrowheads). The frequency of Brp^−^ synapses increases in *unc-104^bris^*; Brp-RNAi and decreases in* unc-104^bris^*; Brp-OE NMJs, however, the availability of Cac-GFP does not follow the same trend. **(B,C)** Quantifications of the percentage of Brp^−^ **(B)** and Cac^−^ **(C)** synapses as shown in **(A)**. **(D–G)** Quantifications of the percentages of Brp^+^Cac^+^ (magenta arrowheads in **A,D**), Brp^+^Cac^−^ (green arrowheads in **A,E**), Brp^−^Cac^+^ (blue arrowheads in **A,F**) and Brp^−^Cac^−^ (yellow arrowheads in **A,G**) synapses. Genotypes in **(A)**: *unc-104^bris^* (*elavX-Gal4/+;unc-104^bris^*/unc-104^d11024^;UAS-Cac-GFP/^+^), *unc-104^bri^*;Brp-RNAi (*elavX-Gal4/+*;*unc-104^bris^*/unc-104^d11024^;*UAS-Brp-RNAi/UAS-Cac-GFP*), *unc-104^bri^*;Brp-OE (*elavX-Gal4/+*;*unc-104^bris^*/unc-104^d11024^;*UAS-Brp/UAS-Cac-GFP*). Experiments were performed at 29°C. Scale bar: 2 μm. Statistical test: one-way ANOVA followed by Tukey’s Multiple Comparison Test. *p < 0.05; **p < 0.01; ***p < 0.001; n.s. *p* > 0.05. Error bars represent SEM.

To further examine the pattern of Brp and Cac localization at *unc-104^bris^* mutant synapses, we manipulated Brp quantity in the *unc-104^bris^* genetic background with either RNAi-mediated knockdown or ectopic overexpression. Brp knockdown in the *unc-104^bris^* background approximately doubled the fraction of Brp^−^ synapses, resulting in ~60% of postsynaptic density (PSD) unapposed by presynaptic Brp puncta (Figure 3B); however, no change in the percentage of Cac^−^ synapses between *unc-104^bris^* and *unc-104^bris^*;Brp-RNAi larvae was observed (25.8% vs. 23.5%, *p* > 0.05; Figure 3C). Brp overexpression in *unc-104^bris^* larvae led to the formation of more presynaptic Brp puncta and decreased the percentage of Brp^−^ synapses in *unc-104^bris^*;Brp-OE (Figure 3B). There was also a mild decrease in Cac^−^ synapses upon Brp overexpression (Figure 3C).

The sharp increase of Brp^−^ synapses in *unc-104^bris^*;Brp-RNAi lead to a reduction in the percentage of Brp^+^Cac^+^ (Figure 3A, magenta arrowheads) synapses in *unc-104^bris^*;Brp-RNAi compared to *unc-104^bris^* and *unc-104^bris^*;Brp-OE (Figure 3D). In *unc-104^bris^*;Brp-RNAi larvae, there was a dramatic increase in Brp^−^Cac^+^ synapses compared with *unc-104^bris^* mutants (~3% in *unc-104^bris^* vs. ~39% in *unc-104^bris^*;Brp-RNAi, *p* < 0.001; Figure 3A, blue arrowheads and Figure 3F). This was likely due to the large number of otherwise normal AZs in *unc-104^bris^* mutants losing Brp as a result of RNAi-mediated knockdown. It is noteworthy that these Cac puncta at Brp^−^ synapses in *unc-104^bris^*;Brp-RNAi larvae seemed smaller than those at Brp^+^ synapses (Figure 3A, blue vs. magenta arrowheads), confirming that Brp is, as previously described (Kittel et al., 2006), important for ensuring high Cac density but not mandatory for Cac localization to AZs. Compared to *unc-104^bris^* mutants, there was a three-fold increase in the percentage of Brp^+^Cac^−^ synapses at* unc-104^bris^*;Brp-OE NMJs (Figure 3A, green arrowheads and Figure 3E), likely because the elevated Brp quantity enhanced Brp nucleation in the otherwise nascent AZ, but the Brp puncta at these locations were not able to efficiently recruit Cac. Therefore, our data suggest that the previously reported temporal order that Cac is integrated into AZs prior to Brp is not mandatory (Fouquet et al., 2009). Consistently, the percentage of Brp^−^Cac^−^ synapses was dramatically reduced in the *unc-104^bris^*, Brp-OE group (Figures 3A,G, yellow arrowheads). Brp knockdown in the *unc-104^bris^* mutant background did not change the amount of Brp^−^Cac^−^ synapses (Figure 3G), suggesting that reduction of Brp at *unc-104^bris^* mutant NMJs did not affect the localization of Cac at the subset of synapses that would otherwise form normal AZs.

### Impaired Anterograde Axonal Transport in *unc-104^bris^* Mutants

**Figure 4:**
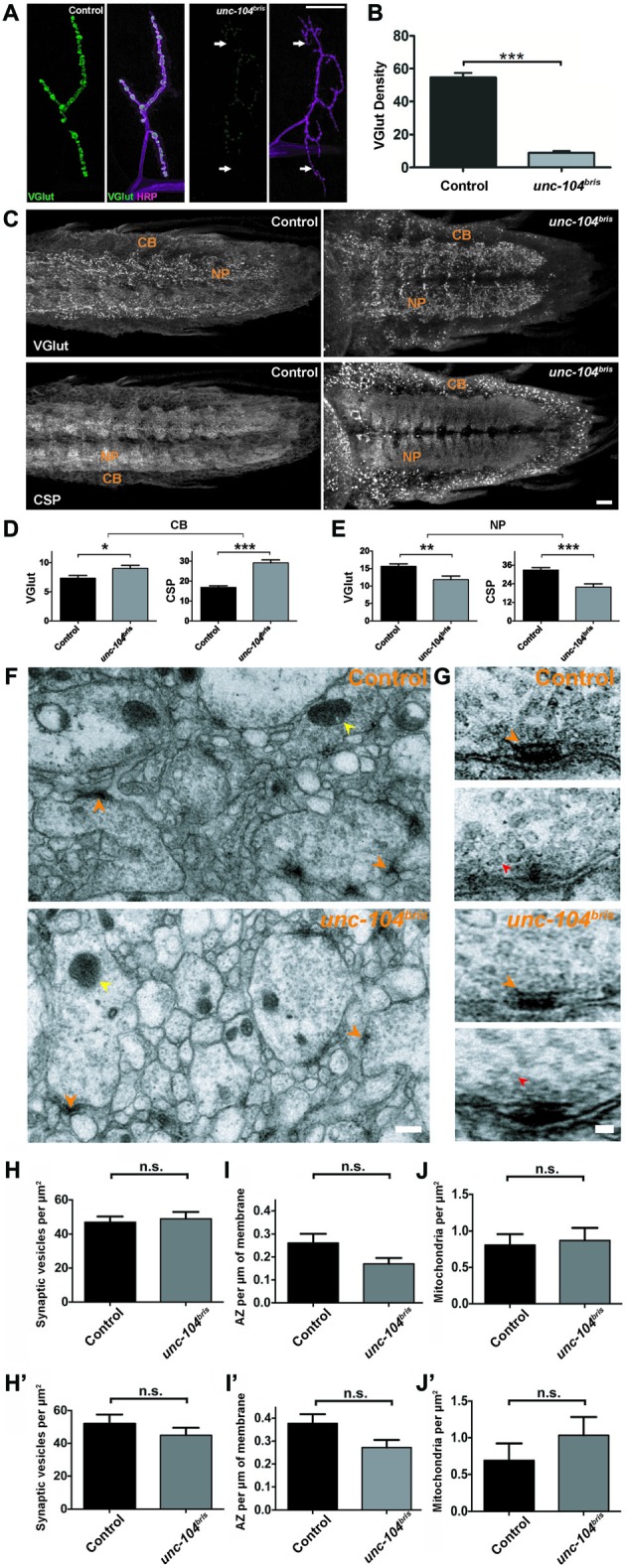
**Characterization of impaired transport at the NMJ and ventral nerve cord (VNC) in *unc-104^bris^* mutants. (A)** Representative confocal images of NMJs from control and *unc-104^bris^* mutant larvae stained with antibody against vescicular glutamate transporter (VGlut, green in **A**). Neuronal membranes were visualized using horseradish peroxidase (HRP) staining (magenta in **A**). **(B)** Quantification of VGlut at the NMJs shown in **(A)**, per HRP area (VGlut density; control: 54.70 ± 2.75 a.u.; *unc-104^bris^*: 8.92 ± 1.04 a.u., *p* < 0.001). Experiments were performed at 29°C. **(C)** Representative confocal images showing abnormal accumulation of VGlut and cysteine string protein (CSP) in the cell body (CB) region of the larval VNC in *unc-104^bris^* compared to control larvae. **(D,E)** Quantifications of the intensities of VGlut and CSP (in a.u.) in the CB and neuropil (NP) region of the VNC in control and *unc-104^bris^* larvae. VGlut intensity at the CB (control: 7.37 ± 1.40 a.u.; *unc-104^bris^*: 9.017 ± 1.71 a.u., *p* < 0.05), CSP intensity at the CB (control: 16.91 ± 2.46 a.u.; *unc-104^bris^*: 29.117 ± 4.80 a.u., *p* < 0.001), VGlut intensity at the NP (control: 15.61 ± 2.25 a.u.; *unc-104^bris^*: 11.79 ± 3.46 a.u., *p* < 0.01), CSP intensity at the NP (control: 32.85 ± 4.77 a.u.; *unc-104^bris^*: 21.865 ± 6.58 a.u., *p* < 0.001). **(F,G)** Representative electron micrographs showing the NP region of VNCs from control and *unc-104^bris^* larvae **(F)** and two representative T-bars from control and *unc-104^bris^* mutant larval VNC each **(G)**. **(H–J)** Quantifications of synaptic vesicles (SVs; red arrowheads), AZs (orange arrowheads) and mitochondria (yellow arrowheads) at the VNCs in control and *unc-104^bris^* larvae in **(F)**. *n* > 40, *n* represents number of cells analyzed. **(H′–J′)** Quantifications of SVs, AZs and mitochondria at the VNCs in control and *unc-104^bris^* larvae with analysis only limited to boutons with the presence of a synapse. *n* > 40, *n* represents number of cells analyzed. Genotypes: control (*w^1118^*), *unc-104^bris^* (;*unc-104^bris^*/unc-104^d11024^;). Scale bar in **(A)**: 10 μm, **(C)**: 10 μm, **(F)**: 0.3 μm, **(G)**: 0.1 μm. Experiments were performed at 25°C. Statistical test: Student’s *t* test. *p < 0.05; **p < 0.01; ***p < 0.001; n.s. *p* > 0.05. Error bars indicate the SEM.

Given the defective AZ maturation observed in *unc-104^bris^*, we addressed whether the mutation affected SV abundance and localization within the NMJ. Previously, Pack-Chung et al. (2007) described a redistribution of *Drosophila* VGlut (Daniels et al., 2004, 2006; Mahr and Aberle, 2006) within the VNC of *unc-104* null mutant embryos. While VGlut signal was increased in the CB–rich area of the cortex, less was detected in the neuropil (NP; Pack-Chung et al., 2007). This result suggests an impairment of SV transport. Thus, we investigated the intensity of VGlut at the NMJ in *unc-104^bris^* mutant larvae. Whereas in control NMJs intense VGlut signal marked the presence of SVs filling entire synaptic boutons, *unc-104^bris^* mutant NMJs had much thinner branches and smaller boutons and showed a severe reduction in VGlut staining at most parts of the NMJ. VGlut was undetectable at some distal segments where the NMJ was particularly thin (Figure 4A, arrows; Figure 4B).

The reduction of VGlut from the NMJ of *unc-104^bris^* mutants might be due to a transport defect, or be caused by the inability of SVs to be stabilized in boutons that are largely devoid of AZs since *cac* null mutant embryos have been shown to have a reduced releasable vesicle pool (Hou et al., 2008). Furthermore, we cannot exclude that the reduced staining is a result of reduced abundance of VGlut protein per SV, rather than being indicative of a reduction in the number of SVs. To address this issue, we investigated the putative reduction of SVs using both VGlut and an additional marker cysteine string protein (CSP) in another model, synapses in the larval NP (Zinsmaier et al., 1990, 1994). Indeed, the levels of VGlut and CSP at the NP region of the VNC were also reduced in *unc-104^bris^* mutants (Figures 4C,E), indicating that the number of SVs is reduced at these synapses. Reminiscent of the elevated levels of Brp reported in the CB region of the VNC in *unc-104^bris^* mutants (Kern et al., 2013), VGlut and CSP levels were enhanced at the CB region in *unc-104^bris^* larvae compared to the control (Figures 4C,D). This phenotype has previously been interpreted as indicative for impaired initiation of axonal transport (Pack-Chung et al., 2007; Kern et al., 2013).

The accumulation of VGlut and CSP signal at the CB region of VNC in *unc-104^bris^* mutants together with reduced signal in the NP might be indicative of fewer SVs at synapses. To test this hypothesis, we performed ultrastructural analysis and quantified the abundance of SVs in the NP region of the VNC in wild-type and *unc-104^bris^* mutants. Our analysis did not reveal any significant differences in the number of SVs between the control and *unc-104^bris^* in the NP (Figures 4F–H; red arrowheads). Next, we quantified the number of AZs at the NP in *unc-104^bris^* mutants. The number of AZs at these synapses revealed a trend towards being reduced, but the difference was not statistically significant (Figures 4F,G,I, orange arrowheads, *p* = 0.056). We also quantified the abundance of mitochondria at the NP of* unc-104^bris^* animals and observed no difference compared with controls (Figures 4F,G,J; yellow arrowheads). To rule out the possibility that the size of the neurite cross sections analyzed could preferentially affect our quantifications, we limited our analysis to only those neurite cross sections that had an AZ. These data also did not reveal any difference in the number of SVs, AZs or mitochondria between control and *unc-104^bris^* mutants (Figures 4H′–J′).

**Figure 5:**
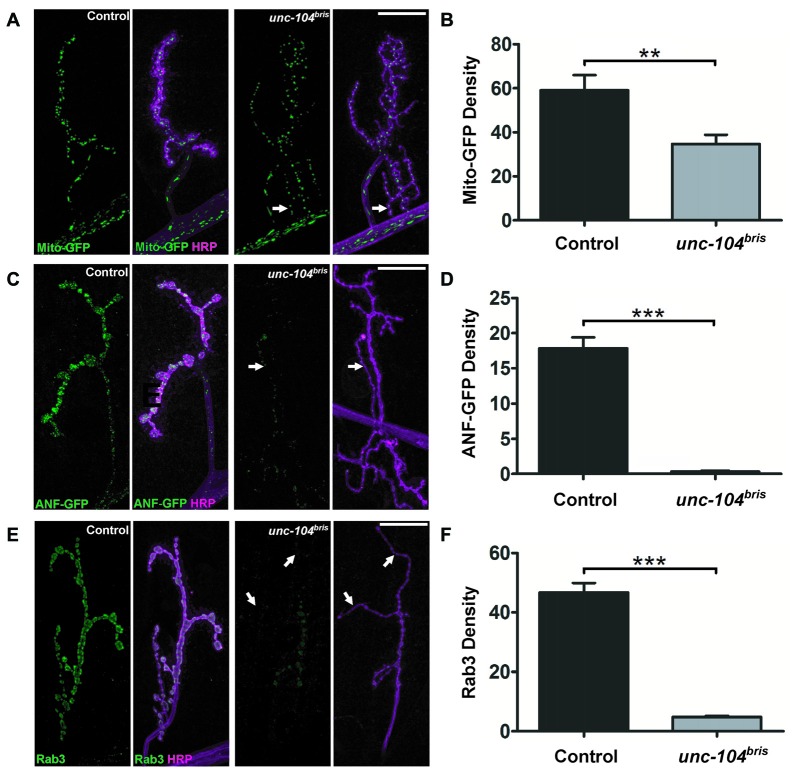
**Synaptic abundance of different cargoes at the NMJ of *unc-104^bris^* mutants.** Confocal images of NMJs in control and *unc-104^bris^* mutant larvae expressing mito-GFP (green, in **A**) or dense core vesicle (DCV) marker GFP tagged atrial natriuretic factor (ANF-GFP; green, in **C**) induced by the *D42-Gal4* driver. **(A)** mito-GFP localization was only mildly affected in* unc-104^bris^* mutant NMJs (per HRP area; Mito density). **(C)** In contrast, ANF-GFP was barely present in* unc-104^bris^* mutant NMJs (per HRP area; ANF-GFP density) compared to control NMJs (per HRP area). Quantifications (in a.u.) of mito-GFP **(B)** and ANF-GFP **(D)** per HRP area respectively as seen in **(A,C)**. Mito-GFP abundance in *unc-104^bris^* mutant NMJs decreased slightly (control: 59.09 ± 6.84 a.u.; *unc-104^bris^*: 34.77 ± 4.13 a.u., *p* < 0.01) whereas ANF-GFP in *unc-104^bris^* mutant NMJs dropped to only 2% of control (control: 17.84 ± 1.56 a.u.; *unc-104^bris^*: 0.34 ± 0.10 a.u., *p* < 0.001). **(E)** Immunostaining of Rab3 (green in **E**) reveals that its quantity also decreased significantly at *unc-104^bris^* mutant NMJs. **(F)** Quantification of Rab3 at the NMJs (per HRP area; Rab3 density) shown in **(E)** in a.u. (control: 46.69 ± 3.25 a.u.; *unc-104^bris^*: 4.77 ± 0.46 a.u., *p* < 0.001). *N* = 9–13 NMJs in each group. Genotypes: in **(A,B)** control (;;*D42-Gal4, UAS-mito-GFP/+*), *unc-104^bris^* (;*unc-104^bris^*/unc-104^d11024^;*D42-Gal4, UAS-mito-GFP/+*); **(C,D)** control (;;*D42-Gal4, UAS-anf-GFP/+*), *unc-104^bris^* (;*unc-104^bris^*/unc-104^d11024^;*D42-Gal4, UAS-ANF-GFP/+*); **(E,F)** control (*w1118*), *unc-104^bris^* (;*unc-104^bris^*/unc-104^d11024^;). Experiments were performed at 29°C. Scale bars: 10 μm. Statistical test: Student’s *t* test. **p < 0.01; ***p < 0.001. Error bars represent SEM.

While depletion of synaptic mitochondria has been associated with synapse destabilization in the context of impaired kinesin-1 based transport (Fuger et al., 2012), no defects in mitochondrial transport have been observed in the *unc-104* null mutant (Pack-Chung et al., 2007). Consistently, ultrastructural analysis provided no evidence of a difference between control and *unc-104^bris^* in the number of mitochondria in neurites of the larval NP (Figures 4F,G,J,J′). This finding suggests that there is no overall breakdown of axonal transport occurring in *unc-104^bris^* larvae. To further test this hypothesis, we investigated mitochondrial abundance at the larval NMJs using mito-GFP. We observed a moderate reduction of mitochondrial density in *unc-104^bris^* mutants compared to the controls, which might reflect a reduced demand for ATP due to less active synapses at mutant NMJs (Figures 5A,B).

We next examined the localization of DCVs at the NMJ using ANF-GFP transgenes (Rao et al., 2001). ANF-GFP positive DCVs were abundant in wildtype NMJs, while in *unc-104^bris^* mutant NMJs the signal was severely diminished, dropping to ~2% of wildtype (Figures 5C,D). We proceeded to examine the localization of Rab3, a SV associated protein previously shown to be an important regulator of synaptic maturation (Graf et al., 2009; Chen et al., 2015), which is also kinesin-3 cargo (Ghila and Gomez, 2008; Niwa et al., 2008). We observed a strong, ~10-fold reduction of Rab3 immunoreactivity at *unc-104^bris^* mutant NMJs (Figure 5E arrows and Figure 5F). Considering the importance of Rab3 in AZ nucleation (Graf et al., 2009), some aspects of impaired AZ formation and maturation in *unc-104^bris^* mutants might be due to synaptic loss of Rab3. In summary, we observe a selective impairment in kinesin-3 based transport, rather than a general breakdown of axonal transport, even late into the pathogenesis.

### Rab3 Ameliorates NMJ But not VNC Transport Phenotypes in Mutant *unc-104^bris^*

**Figure 6:**
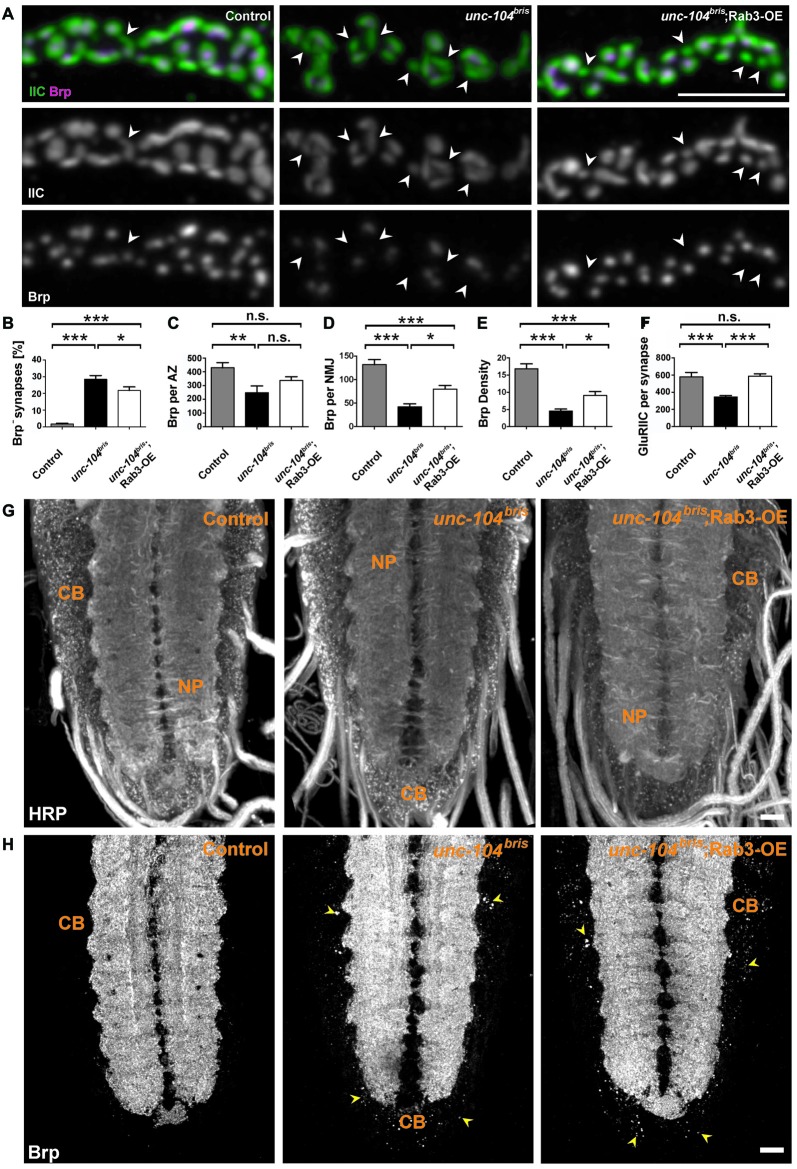
**Rab3 ectopic expression partially ameliorates *unc-104^bris^* mutant phenotypes at the NMJ but not VNC phenotype. (A)** A stretch of synaptic boutons at the NMJ costained with antibodies against GluRIIC (green in upper panel, gray in 2nd panel) and Brp (magenta in upper panel, gray in 3rd panel). **(B–F)** Quantifications of percentage Brp^−^ synapses **(B)** and Brp content per Brp^+^ AZ (in a.u.; **C**) reveal that the percentage of Brp^−^ synapses decreases upon Rab3 expression and the size of each Brp puncta remains unchanged. Brp content per NMJ (in a.u.; **D**) as well as the Brp density (in a.u.; Brp content normalized by HRP area) **(E)** increase following Rab3 expression. Expression of Rab3 fully rescues GluRIIC levels (in a.u.) at postsynaptic density (PSD) of *unc-104^bris^* mutant NMJs to control levels **(F)**. **(G,H)** Representative confocal images from larval VNC stained with antibodies against HRP **(G)** and Brp **(H)**. **(H)** Abnormal accumulation of Brp in the cell CB of the VNC in *unc-104^bris^* mutant compared to the control. Overexpression of Rab3 in *unc-104^bris^* background does not reduce accumulation of Brp at the CB region (indicated by yellow arrowheads). Scale bar in **(A)**: 5 μm, **(G)**: 15 μm. Genotypes: control (*elavX-Gal4/+*;;), *unc-104^bris^* (*elavX-Gal4/+*;*unc-104^bris^*/unc-104^d11024^;), *unc-104^bri^*;Rab3-OE (*elavX-Gal4/+*;*unc-104^bris^*/unc-104^d11024^;*UAS-Rab3/+*). Experiments were performed at 29°C. Statistical test: one-way ANOVA followed by Tukey’s Multiple Comparison Test. *p < 0.05; **p < 0.01; ***p < 0.001; n.s., *p* > 0.05. Error bars indicate the SEM.

Given the role of Rab3 in local AZ protein distribution (Graf et al., 2009; Chen et al., 2015), we hypothesized that the failure to enrich Rab3 at the NMJ in *unc-104^bris^* mutants could contribute to the synapse maturation defects in these mutants. To test this hypothesis, we expressed a Rab3 transgene and examined the percentage of Brp^−^ synapses by immunostaining at the larval NMJ. Indeed, ectopic overexpression of Rab3 in the *unc-104^bris^* genetic background partially rescued the percentage of Brp^−^ AZs per NMJ (Figures 6A,B). In *rab3* mutants, defects in the nucleation of AZ assembly have been proposed as a cause for the formation of many Brp^−^ negative synapses (Graf et al., 2009). At the same time, residual AZs are much larger and incorporate much more Brp than control synapses (Graf et al., 2009). To address whether a similar mechanism causes the Brp^−^ synapses observed in *unc-104^bris^* mutants, the amount of Brp per Brp^+^ AZ (Brp per AZ) was quantified. In contrast to *rab3* mutants, Brp per Brp^+^ AZ is not increased in *unc-104^bris^* mutants (Figures 6A,C). Moreover, the amount of Brp at the NMJ is, as previously described, reduced (Figures 6A–E; Kern et al., 2013). Interestingly, overexpression of Rab3 does not reduce average Brp puncta size in the *unc-104^bris^* mutant background (Figures 6A,C), as would be expected if the function of Rab3 in nucleating was crucial in the *unc-104^bris^* NMJ. To the contrary, we observe a trend towards a larger Brp puncta size in *unc-104^bris^*;Rab3-OE animals compared to *unc-104^bris^* larvae, however this difference was not statistically significant (Figures 6A,C). Given the trend of an increase of Brp per AZ, we asked whether Rab3 might affect the overall Brp abundance. To examine this possibility we quantified total Brp per NMJ and Brp density (Brp per μm^2^ HRP positive NMJ area). Our quantifications reveal that indeed, both Brp per NMJ and Brp density were increased in *unc-104^bris^*;Rab3-OE larvae compared to *unc-104^bris^* (Figures 6A,D,E). However, the efficiency of the rescue was incomplete as the level of Brp is still higher in control animals. Similar to the observation that expression of Brp in *unc-104^bris^* background increases Brp levels at the NMJ without altering the overall NMJ morphology in *unc-104^bris^* (Kern et al., 2013), expression of Rab3 had no obvious effect on the overall NMJ morphology (data not shown). We previously reported that *unc-104^bris^* NMJs are characterized by a reduction in the amount of GluRIIC at the PSD (Kern et al., 2013). Interestingly, overexpression of Rab3 also restored the abundance of GluRIIC per PSD at *unc-104^bris^* NMJs (Figures 6A,F).

Next, we sought to elucidate whether Rab3 rescues defects at the level of axonal transport initiation or rather locally at synapses. Thus, we examined whether Rab3 also reversed the ectopic accumulation of transport cargo in the CBs of the motoneurons. Ectopic expression of Rab3 does not ameliorate the abnormal accumulation of Brp in the CB region of the larval VNC (Figures 6G,H; yellow arrowheads), suggesting that Rab3 likely rescues NMJ phenotypes downstream of *unc-104^bris^* by a mechanism that acts locally at the NMJ.

## Discussion

### Clinical and Cellular Consequences of Impaired *KIF1A* Function

Recent studies have led to the identification of several mutations in *KIF1A*. An autosomal recessive mode of inheritance has been implicated in patients affected by the neurodegenerative diseases HSAN2 and SPG30 (Erlich et al., 2011; Klebe et al., 2012). HSAN2 is characterized by peripheral nerve degeneration of sensory neurons that results in a loss of sensory perception and variable degrees of autonomic dysfunction (Axelrod, 2002; Hilz, 2002). For example, a one base pair deletion downstream of the motor domain of KIF1A was identified in a homozygous state in three families affected by HSAN2 (Rivière et al., 2011). Frameshift mutations in HSAN2 cause severe distal sensory loss and distal muscle weakness with an age of onset varying from congenital to 15 years of age (Rivière et al., 2011).

SPG30, a subtype of hereditary spastic paraplegia (HSP), is a clinically and genetically heterogeneous motoneuron disease characterized by progressive stiffness and weakness of lower limbs caused by axonopathy of corticospinal tract neurons (Deluca et al., 2004; Lo Giudice et al., 2014). Substitution A255V at the highly conserved residue of the motor domain was reported in three siblings of the same family affected by SPG30 who were homozygous for the mutation, whereas both unaffected parents and siblings were heterozygous for this mutation (Erlich et al., 2011). Moreover Klebe et al. (2012) identified a homozygous R350G substitution and the previously described A255V variants in the motor domain of KIF1A in SPG30 patients.

A putatively hypermorphic *de novo* T99M variant, in the highly conserved P loop of the ATP binding site of the motor domain of KIF1A has been identified in a patient with ID (Hamdan et al., 2011), spasticity and axial hypotonia and in a second patient with a more severe phenotype including optic atrophy, growth failure and progressive cerebellar atrophy, in addition to ID (Citterio et al., 2015). How different mutation types in *KIF1A* alter protein function remains unclear, but may be related to a residual function of the protein in the case of haploinsufficiency or the toxic gain-of-function and dominant negative effects that impair endogenous protein function. *Drosophila* has been utilized successfully to investigate dominant negative functions of KIF5A (Fuger et al., 2012) and would be an ideal organism to test the functional implications of mutations in conserved domains of unc-104.

The clinical manifestations of diseases associated with impaired *KIF1A* function are very broad, highlighting that *KIF1A* is important for various cellular and molecular processes. Neurons primarily affected in SPG30 and HSAN2 patients possess long axons, making kinesin-based intracellular anterograde transport imperative. Functional *in vitro* validation of the T99M mutation (KIF1A^T99M^) revealed reduced distal localization of KIF1A^T99M^ variant in transfected primary rat hippocampal neurons and increased accumulation at the CBs and proximal neurons compared to wildtype KIF1A (Esmaeeli Nieh et al., 2015). In addition, microtubule gliding assays of KIF1A^T99M^ on glass coverslips under saturating ATP conditions showed no motility unlike the control wildtype KIF1A (Esmaeeli Nieh et al., 2015).

In *Drosophila* and mice, *unc*-*104/KIF1A* null animals die at the late embryonic stage or shortly after birth, respectively (Yonekawa et al., 1998; Pack-Chung et al., 2007). Null mutant *Drosophila* embryos demonstrate nerve out-growth but fail to mature synaptic boutons and suffer from an almost complete loss of SVs and AZs at the NMJ (Pack-Chung et al., 2007). Likewise, loss of *KIF1A* in mice result in a specific impairment of SV precursor transport resulting in reduced SV density, and clusters of clear small vesicles accumulate in the CBs of motoneurons (Yonekawa et al., 1998). Although these studies highlight the importance of kinesin-3 mediated transport, the early lethality associated with complete loss of *KIF1A* function prevents a more detailed analysis of symptoms associated with a chronic, partial loss of kinesin-3 function.

By employing a hypomorphic allele of the *Drosophila unc-104, unc-104^bris^*, caused by a point mutation in the FHA domain of Unc-104, we have previously shown that loss-of-function of the kinesin-3 motor disrupts synaptic bouton and AZ development at the *Drosophila* NMJ (Kern et al., 2013). Intriguingly, these larvae lacked some of the typical features associated with neuropathy caused by putative length-dependent impairment in axonal transport such as axonal swellings and progressive posterior paralysis (Barkus et al., 2008; Kern et al., 2013). While terminal atrophy has been associated with a different* unc-104* allele (Barkus et al., 2008) we previously did not find evidence for synapse retraction in *unc-104^bris^* mutant larvae (Kern et al., 2013). Herein, a more detailed analysis of NMJs provides evidence for a severe reduction in the amount of SV-associated proteins at distal boutons while showing no obvious signs of a classical dying-back pathology in *unc-104^bris^* mutant larvae. Using a different allele, Barkus et al. (2008) described major impairments in the anterograde fast transport of DCVs and SVs. Moreover, they suggested that Unc-104 has little contribution in the anterograde transport of mitochondria. We could further confirm that similar effects are observed in *unc-104^bris^* larvae. While proper Unc-104 function seems to be imperative for synaptic localization of the SV proteins VGlut (Figures 4A–E), CSP (Figures 4C–E) the DCV marker ANF-GFP (Figures 5C,D), Rab3 (Figures 5E,F), and the AZ components Liprin-α (Figure 1B) and Cac (Figures 2, 3), it seems to have only a minor contribution to the anterograde transport of mitochondria (Figures 5A,B).

### Localization of Synaptic Proteins in *unc-104^bris^* and Brp-RNAi NMJs

This study reveals that AZ proteins SRPK79D, Liprin-α and Cac are absent from Brp^−^ synapses in *unc-104^bris^* mutant NMJs, suggesting these presynaptic sites are likely devoid of the CAZ (Figures 1, 2). Kinesin-3 has been previously implicated in the transport of Brp (Pack-Chung et al., 2007; Kern et al., 2013). While stalling of synapse maturation could be explained simply by a lack of availability of AZ components, we feel that the underlying mechanisms are likely more complex. The current study characterizes defects in the distribution of AZ proteins to individual release sites. SRPK79D fails to localize at Brp^−^ synapses in NMJs of both *unc-104^bris^* mutant and Brp-RNAi expressing larvae, suggesting a role for Brp in recruiting SRPK79D to the AZ (Figure 1A). Previous studies have shown that loss-of-function of *srpk79D* causes impaired axonal localization of Brp (Johnson et al., 2009; Nieratschker et al., 2009) and reduced localization of Brp in nerve terminals (Johnson et al., 2009). In this study, we show a reciprocal impairment of SRPK79D localization at the NMJ in *unc-104^bris^* and Brp-RNAi larvae (Figure 1A). In contrast, although Cac and Liprin-α persist in Brp^−^ synapses in Brp-RNAi larvae, they are absent from the Brp^−^ synapses in* unc-104^bris^* mutants (Figures 1B, 2A). Comparison between* unc-104^bris^* mutants and Brp-RNAi larvae suggests that the synapse maturation phenotype in *unc-104^bris^* mutants is not downstream of loss of Brp from a subset of AZs. Consistently, it has been shown that during synapse maturation, both Cac and Liprin-α precede the formation of Brp puncta (Fouquet et al., 2009), and that Brp localizes to AZs only ~3 h after the incorporation of GluR into the PSD (Rasse et al., 2005). The fact that modulating Brp levels at the NMJ in *unc-104^bris^* by RNAi or by ectopic expression does not modify the percentage of Cac^−^ synapses drastically is in line with the temporal recruitment of Cac prior to Brp at the AZ (Figures 3A–C).

The Brp^−^ synapses in Brp-RNAi larvae did not exhibit obvious Liprin-α localization defects, suggesting that localization of Liprin-α at AZs is not dependent on Brp (Figure 1B). Liprin-α regulates the size and shape of Brp clusters located at the AZ (Fouquet et al., 2009). Our result indicates that Brp does not have an obvious reciprocal effect on Liprin-α localization. Intriguingly, Liprin-α has been shown to have an evolutionarily conserved association with kinesin-3. It has been suggested that Liprin-α functions as a KIF1A receptor, linking the motor protein to its cargo for transport in neurons (Shin et al., 2003). The mutation in *unc-104^bris^* within the FHA domain may disrupt its ability to bind to Liprin-α, which could contribute to the synaptic phenotype in *unc-104^bris^* mutants. Consistent with previous reports showing reduced Cac localization in *brp* mutants (Kittel et al., 2006), Brp^−^ synapses at the NMJs of Brp-RNAi expressing larvae have less Cac compared to Brp^+^ synapses (Figure 2A). The C-terminus of Cac has been shown to physically interact with the N-terminus of Brp (Fouquet et al., 2009). This interaction has been suggested to be crucial for the continued incorporation of Cac during synapse maturation, but not the initial Cac localization in nascent AZs (Fouquet et al., 2009). In *unc-104^bris^* mutants, Brp^−^ presynaptic sites do not contain any detectable Liprin-α or Cac (Figures 1B, 2A), suggesting that the AZ maturation is likely stalled at a very early stage at these sites.

### Apparent Normal AZ Formation at a Subset of Synapses in *unc-104^bris^*

Despite an obvious increase in Brp^−^ synapses in *unc-104^bris^* mutants, ~75% of the synapses at *unc-104^bris^* mutant NMJs are Brp^+^. Manipulation of Brp levels revealed that although a subset of the synapses seem to be devoid of presynaptic AZs, the rest of the synapses largely have normal AZs. Knockdown of Brp resulted in more Brp^−^ synapses in *unc-104^bris^* mutants, but no change in Cac^−^ synapses (Figures 3A–C). The dramatic increase of Brp^−^Cac^+^ synapses in *unc-104^bris^*;Brp-RNAi animals is coincidental with the reduction of Brp^+^Cac^+^ synapses (Figures 3D,F). This suggests that these synapses are able to retain a low level of Cac akin to those in Brp-RNAi larvae in otherwise wildtype background (Figure 2A). On the other hand, overexpression of Brp in the *unc-104^bris^* background decreased the number of Brp^−^ synapses, suggesting that AZs in *unc-104^bris^* are capable of Brp clustering, but fail to mature by continuing to incorporate core components. Either due to the lack of other AZ machinery, absence of Rab3-mediated coordination of AZ maturation, or due to competition with other synapses, these Brp^+^ AZs fail to recruit Cac. Consistently, there is an increase of Brp^+^Cac^−^ synapses and no change in Brp^+^Cac^+^ synapses in the *unc-104^bris^*;Brp-OE group (Figures 3D,E).

### Role of Rab3 in the Maturation of AZ

We show that transport of Rab3 is severely impaired in *unc-104^bris^* mutants (Figures 5E,F). Rab3 has been shown to be an important regulator of AZ formation, primarily due to its function in promoting AZ nucleation and the distribution of AZ components at the NMJ (Giagtzoglou et al., 2009; Graf et al., 2009; Chen et al., 2015). Ectopic Rab3 expression in *unc-104^bris^* background reduces the number of Brp^−^ synapses (Figures 6A,B). If Rab3 decreases the Brp^−^ synapse number simply by enhancing the probability that an AZ forms at the NMJ, we would expect a further decrease in the average Brp puncta size accompanying the decreased Brp^−^ synapses. Instead, we observe a trend of increased Brp per AZ and a significant increase of Brp per NMJ upon expression of Rab3 in the *unc-104^bris^* mutant NMJs compared to the *unc-104^bris^* mutant alone (Figures 6C,D). Furthermore, while *rab3* mutants have a significant increase of Brp per residual Brp^+^ AZ, we observe no increase in the level of Brp per Brp^+^ AZ in *unc-104^bris^* mutant larvae. Therefore, while Rab3 overexpression partially rescues the Brp^−^ synapses in *unc-104^bris^* mutant NMJs, this effect is likely not solely through its function of enhancing AZ nucleation. It was previously reported that increasing Brp quantity at the NMJ by overexpressing a UAS-Brp transgene could partially rescue the Brp^−^ synapses in *unc-104^bris^* mutants (Kern et al., 2013), so the reduced Brp^−^ synapses in *unc-104^bris^*;Rab3-OE larvae could be entirely a secondary effect of increased Brp quantity at the NMJ, the mechanism of which is still unclear.

One attractive explanation for the increased Brp quantity at the NMJ in *unc-104^bris^*;Rab3-OE larvae would be that Rab3 may promote initiation of the transport of AZ components. However, we did not observe any changes in the accumulation of synaptic materials in the VNC cell bodies in *unc-104^bris^*;Rab3-OE (Figure 6G). While we lack evidence for the underlying mechanism, it is tempting to speculate that Rab3 overexpression might affect the activity-dependent capture of retrograde cargo in a mechanism similar to the capture of transiting peptidergic vesicles (Shakiryanova et al., 2006), which are likewise strongly reduced (Figures 5C,D) at *unc-104^bris^* mutant NMJs.

## Conclusion

In summary, we investigated the cellular phenotypes of impaired intracellular transport caused by a *bris* hypomorphic mutation in *unc-104*. We previously reported an increased percentage of synapses that lack presynaptic Brp in *unc-104^bris^* mutants (Kern et al., 2013). Here, we provide further evidence showing that these synapses are also devoid of other important AZ proteins, implying a generalized failure to form or to mature AZ structures at these presynaptic sites. Synaptic localization of Rab3, a small GTPase previously implicated in regulating *Drosophila* AZ protein incorporation, is severely impaired in *unc-104^bris^* mutants, along with SV and DCV markers. Interestingly, ectopic expression of Rab3 partially rescues synaptic Brp localization in *unc-104^bris^* mutants, but not the ectopic accumulation of Brp in the CB region of the affected motoneurons, suggesting that increasing Rab3 primarily acts locally at the NMJ.

## Author Contributions

TMR: conceived and supervised the project; YVZ and TMR: designed experiments; YVZ, SBH, ZAS, JVK and TMR: performed experiments and analyzed the data; YVZ, SBH, TRJ and TMR: wrote the article.

## Conflict of Interest Statement

The authors declare that the research was conducted in the absence of any commercial or financial relationships that could be construed as a potential conflict of interest.

## Abbreviations

ANF-GFP: GFP tagged atrial natriuretic factor
AZ: active zone
Brp: Bruchpilot
Cac: Cacophony
CB: cell body
CAZ: cytomatrix at the active zone
CSP: cysteine-string protein
DCVs: dense core vesicles
FHA: forkhead associated
GluRIIC: glutamate receptor subunit IIC
HRP: horseradish peroxidase
HSAN2: hereditary and sensory autonomic neuropathy type 2
HSP: hereditary spastic paraplegia
ID: intellectual disability
NP: neuropil
NMJ: neuromuscular junction
PSD: postsynaptic density
SPG30: hereditary spastic paraplegia subtype 30
SRPK79D: Serine-arginine protein kinase at 79D
SVs: synaptic vesicles
VGlut: vesicular glutamate transporter
VNC: ventral nerve cord
UA: uranyl acetate.

## References

[B1] Ackermann F. Waites C. L. Garner C. C. (2015). Presynaptic active zones in invertebrates and vertebrates. EMBO Rep. 16, 923–938. 10.15252/embr.201540434 26160654 PMC4552486

[B2] Axelrod F. B. (2002). Hereditary sensory and autonomic neuropathies. Familial dysautonomia and other HSANs. Clin. Auton. Res. 12, I2–I14. 10.1007/s102860200013 12102459

[B3] Bae H. Chen S. Roche J. P. Ai M. Wu C. Diantonio A.. (2016). Rab3-GEF controls active zone development at the *Drosophila* neuromuscular junction. eNeuro 3. 10.1523/ENEURO.0031-16.2016 27022630 PMC4791486

[B4] Barkus R. V. Klyachko O. Horiuchi D. Dickson B. J. Saxton W. M. (2008). Identification of an axonal kinesin-3 motor for fast anterograde vesicle transport that facilitates retrograde transport of neuropeptides. Mol. Biol. Cell 19, 274–283. 10.1091/mbc.e07-03-0261 17989365 PMC2174192

[B5] Bruckner J. J. Gratz S. J. Slind J. K. Geske R. R. Cummings A. M. Galindo S. E.. (2012). Fife, a *Drosophila* Piccolo-RIM homolog, promotes active zone organization and neurotransmitter release. J. Neurosci. 32, 17048–17058. 10.1523/JNEUROSCI.3267-12.2012 23197698 PMC3524967

[B6] Bury L. A. Sabo S. L. (2015). Building a terminal: mechanisms of presynaptic development in the CNS. Neuroscientist 22, 372–391. 10.1177/1073858415596131 26208860

[B7] Butzlaff M. Hannan S. B. Karsten P. Lenz S. Ng J. Vossfeldt H.. (2015). Impaired retrograde transport by the dynein/dynactin complex contributes to tau-induced toxicity. Hum. Mol. Genet. 24, 3623–3637. 10.1093/hmg/ddv107 25794683

[B8] Chen S. Gendelman H. K. Roche J. P. Alsharif P. Graf E. R. (2015). Mutational analysis of rab3 function for controlling active zone protein composition at the *Drosophila* neuromuscular junction. PLoS One 10:e0136938. 10.1371/journal.pone.0136938 26317909 PMC4552854

[B9] Citterio A. Arnoldi A. Panzeri E. Merlini L. D’Angelo M. G. Musumeci O.. (2015). Variants in KIF1A gene in dominant and sporadic forms of hereditary spastic paraparesis. J. Neurol. 262, 2684–2690. 10.1007/s00415-015-7899-9 26410750

[B10] Daniels R. W. Collins C. A. Chen K. Gelfand M. V. Featherstone D. E. DiAntonio A. (2006). A single vesicular glutamate transporter is sufficient to fill a synaptic vesicle. Neuron 49, 11–16. 10.1016/j.neuron.2005.11.032 16387635 PMC2248602

[B11] Daniels R. W. Collins C. A. Gelfand M. V. Dant J. Brooks E. S. Krantz D. E.. (2004). Increased expression of the *Drosophila* vesicular glutamate transporter leads to excess glutamate release and a compensatory decrease in quantal content. J. Neurosci. 24, 10466–10474. 10.1523/JNEUROSCI.3001-04.2004 15548661 PMC6730318

[B12] Deluca G. C. Ebers G. C. Esiri M. M. (2004). The extent of axonal loss in the long tracts in hereditary spastic paraplegia. Neuropathol. Appl. Neurobiol. 30, 576–584. 10.1111/j.1365-2990.2004.00587.x 15540998

[B13] Erlich Y. Edvardson S. Hodges E. Zenvirt S. Thekkat P. Shaag A.. (2011). Exome sequencing and disease-network analysis of a single family implicate a mutation in KIF1A in hereditary spastic paraparesis. Genome Res. 21, 658–664. 10.1101/gr.117143.110 21487076 PMC3083082

[B14] Esmaeeli Nieh S. Madou M. R. Sirajuddin M. Fregeau B. McKnight D. Lexa K.. (2015). *De novo* mutations in KIF1A cause progressive encephalopathy and brain atrophy. Ann. Clin. Transl. Neurol. 2, 623–635. 10.1002/acn3.198 26125038 PMC4479523

[B15] Fouquet W. Owald D. Wichmann C. Mertel S. Depner H. Dyba M.. (2009). Maturation of active zone assembly by *Drosophila* Bruchpilot. J. Cell Biol. 186, 129–145. 10.1083/jcb.200812150 19596851 PMC2712991

[B16] Frank C. A. Wang X. Collins C. A. Rodal A. A. Yuan Q. Verstreken P.. (2013). New approaches for studying synaptic development, function and plasticity using *Drosophila* as a model system. J. Neurosci. 33, 17560–17568. 10.1523/JNEUROSCI.3261-13.2013 24198346 PMC3818537

[B17] Fuger P. Sreekumar V. Schule R. Kern J. V. Stanchev D. T. Schneider C. D.. (2012). Spastic paraplegia mutation N256S in the neuronal microtubule motor KIF5A disrupts axonal transport in a *Drosophila* HSP model. PLoS Genet. 8:e1003066. 10.1371/journal.pgen.1003066 23209432 PMC3510046

[B18] Geppert M. Sudhof T. C. (1998). RAB3 and synaptotagmin: the yin and yang of synaptic membrane fusion. Annu. Rev. Neurosci. 21, 75–95. 10.1146/annurev.neuro.21.1.75 9530492

[B19] Ghila L. Gomez M. (2008). The evolutionarily conserved gene LNP-1 is required for synaptic vesicle trafficking and synaptic transmission. Eur. J. Neurosci. 27, 621–630. 10.1111/j.1460-9568.2008.06049.x 18279315

[B20] Giagtzoglou N. Mahoney T. Yao C. K. Bellen H. J. (2009). Rab3 GTPase lands Bruchpilot. Neuron 64, 595–597. 10.1016/j.neuron.2009.11.029 20005815 PMC5687091

[B21] Graf E. R. Daniels R. W. Burgess R. W. Schwarz T. L. DiAntonio A. (2009). Rab3 dynamically controls protein composition at active zones. Neuron 64, 663–677. 10.1016/j.neuron.2009.11.002 20005823 PMC2796257

[B22] Hamdan F. F. Gauthier J. Araki Y. Lin D. T. Yoshizawa Y. Higashi K.. (2011). Excess of *de novo* deleterious mutations in genes associated with glutamatergic systems in nonsyndromic intellectual disability. Am. J. Hum. Genet. 88, 306–316. 10.1016/j.ajhg.2011.02.001 21376300 PMC3059427

[B23] Hilz M. J. (2002). Assessment and evaluation of hereditary sensory and autonomic neuropathies with autonomic and neurophysiological examinations. Clin. Auton. Res. 12, I33–I43. 10.1007/s102860200017 12102461

[B24] Hotchkiss L. Donkervoort S. Leach M. E. Mohassel P. Bharucha-Goebel D. X. Bradley N.. (2016). Novel *de novo* mutations in KIF1A as a cause of hereditary spastic paraplegia with progressive central nervous system involvement. J. Child Neurol. 31, 1114–1119. 10.1177/0883073816639718 27034427 PMC5030766

[B25] Hou J. Tamura T. Kidokoro Y. (2008). Delayed synaptic transmission in *Drosophila* cacophonynull embryos. J. Neurophysiol. 100, 2833–2842. 10.1152/jn.90342.2008 18815348

[B26] Johnson E. L. III Fetter R. D. Davis G. W. (2009). Negative regulation of active zone assembly by a newly identified SR protein kinase. PLoS Biol. 7:e1000193. 10.1371/journal.pbio.1000193 19771148 PMC2737616

[B27] Kaufmann N. DeProto J. Ranjan R. Wan H. Van Vactor D. (2002). *Drosophila* liprin-alpha and the receptor phosphatase Dlar control synapse morphogenesis. Neuron 34, 27–38. 10.1016/s0896-6273(02)00643-8 11931739

[B28] Kawasaki F. Zou B. Xu X. Ordway R. W. (2004). Active zone localization of presynaptic calcium channels encoded by the cacophony locus of *Drosophila*. J. Neurosci. 24, 282–285. 10.1523/jneurosci.3553-03.2004 14715960 PMC6729574

[B29] Kern J. V. Zhang Y. V. Kramer S. Brenman J. E. Rasse T. M. (2013). The kinesin-3, unc-104 regulates dendrite morphogenesis and synaptic development in *Drosophila*. Genetics 195, 59–72. 10.1534/genetics.113.151639 23770702 PMC3761313

[B30] Kittel R. J. Wichmann C. Rasse T. M. Fouquet W. Schmidt M. Schmid A.. (2006). Bruchpilot promotes active zone assembly, Ca^2+^ channel clustering and vesicle release. Science 312, 1051–1054. 10.1126/science.1126308 16614170

[B31] Klebe S. Lossos A. Azzedine H. Mundwiller E. Sheffer R. Gaussen M.. (2012). KIF1A missense mutations in SPG30, an autosomal recessive spastic paraplegia: distinct phenotypes according to the nature of the mutations. Eur. J. Hum. Genet. 20, 645–649. 10.1038/ejhg.2011.261 22258533 PMC3355258

[B32] Lee J. R. Srour M. Kim D. Hamdan F. F. Lim S. H. Brunel-Guitton C.. (2015). De novo mutations in the motor domain of KIF1A cause cognitive impairment, spastic paraparesis, axonal neuropathy and cerebellar atrophy. Hum. Mutat. 36, 69–78. 10.1002/humu.22709 25265257

[B33] Liu K. S. Siebert M. Mertel S. Knoche E. Wegener S. Wichmann C.. (2011). RIM-binding protein, a central part of the active zone, is essential for neurotransmitter release. Science 334, 1565–1569. 10.1126/science.1212991 22174254

[B34] Lo Giudice T. Lombardi F. Santorelli F. M. Kawarai T. Orlacchio A. (2014). Hereditary spastic paraplegia: clinical-genetic characteristics and evolving molecular mechanisms. Exp. Neurol. 261, 518–539. 10.1016/j.expneurol.2014.06.011 24954637

[B35] Mahr A. Aberle H. (2006). The expression pattern of the *Drosophila* vesicular glutamate transporter: a marker protein for motoneurons and glutamatergic centers in the brain. Gene Expr. Patterns 6, 299–309. 10.1016/j.modgep.2005.07.006 16378756

[B36] Müller M. Pym E. C. Tong A. Davis G. W. (2011). Rab3-GAP controls the progression of synaptic homeostasis at a late stage of vesicle release. Neuron 69, 749–762. 10.1016/j.neuron.2011.01.025 21338884 PMC3059509

[B37] Nieratschker V. Schubert A. Jauch M. Bock N. Bucher D. Dippacher S.. (2009). Bruchpilot in ribbon-like axonal agglomerates, behavioral defects and early death in SRPK79D kinase mutants of *Drosophila*. PLoS Genet. 5:e1000700. 10.1371/journal.pgen.1000700 19851455 PMC2759580

[B38] Niwa S. Tanaka Y. Hirokawa N. (2008). KIF1B^β^- and KIF1A-mediated axonal transport of presynaptic regulator Rab3 occurs in a GTP-dependent manner through DENN/MADD. Nat. Cell Biol. 10, 1269–1279. 10.1038/ncb1785 18849981

[B39] Ohba C. Haginoya K. Osaka H. Kubota K. Ishiyama A. Hiraide T.. (2015). De novo KIF1A mutations cause intellectual deficit, cerebellar atrophy, lower limb spasticity and visual disturbance. J. Hum. Genet. 60, 739–742. 10.1038/jhg.2015.108 26354034

[B41] Owald D. Fouquet W. Schmidt M. Wichmann C. Mertel S. Depner H.. (2010). A Syd-1 homologue regulates pre- and postsynaptic maturation in *Drosophila*. J. Cell Biol. 188, 565–579. 10.1083/jcb.200908055 20176924 PMC2828917

[B40] Owald D. Sigrist S. J. (2009). Assembling the presynaptic active zone. Curr. Opin. Neurobiol. 19, 311–318. 10.1016/j.conb.2009.03.003 19395253

[B42] Pack-Chung E. Kurshan P. T. Dickman D. K. Schwarz T. L. (2007). A *Drosophila* kinesin required for synaptic bouton formation and synaptic vesicle transport. Nat. Neurosci. 10, 980–989. 10.1038/nn1936 17643120

[B43] Qin G. Schwarz T. Kittel R. J. Schmid A. Rasse T. M. Kappei D.. (2005). Four different subunits are essential for expressing the synaptic glutamate receptor at neuromuscular junctions of *Drosophila*. J. Neurosci. 25, 3209–3218. 10.1523/jneurosci.4194-04.2005 15788778 PMC6725071

[B44] Rao S. Lang C. Levitan E. S. Deitcher D. L. (2001). Visualization of neuropeptide expression, transport and exocytosis in *Drosophila melanogaster*. J. Neurobiol. 49, 159–172. 10.1002/neu.1072 11745655

[B45] Rasse T. M. Fouquet W. Schmid A. Kittel R. J. Mertel S. Sigrist C. B.. (2005). Glutamate receptor dynamics organizing synapse formation *in vivo*. Nat. Neurosci. 8, 898–905. 10.1038/nn1484 16136672

[B46] Richmond J. E. Broadie K. S. (2002). The synaptic vesicle cycle: exocytosis and endocytosis in *Drosophila* and *C. elegans*. Curr. Opin. Neurobiol. 12, 499–507. 10.1016/s0959-4388(02)00360-4 12367628

[B47] Rivière J. B. Ramalingam S. Lavastre V. Shekarabi M. Holbert S. Lafontaine J.. (2011). KIF1A, an axonal transporter of synaptic vesicles, is mutated in hereditary sensory and autonomic neuropathy type 2. Am. J. Hum. Genet. 89, 219–230. 10.1016/j.ajhg.2011.06.013 21820098 PMC3155159

[B48] Shakiryanova D. Tully A. Levitan E. S. (2006). Activity-dependent synaptic capture of transiting peptidergic vesicles. Nat. Neurosci. 9, 896–900. 10.1038/nn1719 16767091

[B49] Shin H. Wyszynski M. Huh K. H. Valtschanoff J. G. Lee J. R. Ko J.. (2003). Association of the kinesin motor KIF1A with the multimodular protein liprin-alpha. J. Biol. Chem. 278, 11393–11401. 10.1074/jbc.m211874200 12522103

[B50] Südhof T. C. (2012). The presynaptic active zone. Neuron 75, 11–25. 10.1016/j.neuron.2012.06.012 22794257 PMC3743085

[B51] Wagh D. A. Rasse T. M. Asan E. Hofbauer A. Schwenkert I. Durrbeck H.. (2006). Bruchpilot, a protein with homology to ELKS/CAST, is required for structural integrity and function of synaptic active zones in *Drosophila*. Neuron 49, 833–844. 10.1016/j.neuron.2006.02.008 16543132

[B52] Ylikallio E. Kim D. Isohanni P. Auranen M. Kim E. Lonnqvist T.. (2015). Dominant transmission of *de novo* KIF1A motor domain variant underlying pure spastic paraplegia. Eur. J. Hum. Genet. 23, 1427–1430. 10.1038/ejhg.2014.297 25585697 PMC4592090

[B53] Yonekawa Y. Harada A. Okada Y. Funakoshi T. Kanai Y. Takei Y.. (1998). Defect in synaptic vesicle precursor transport and neuronal cell death in *KIF1A* motor protein-deficient mice. J. Cell Biol. 141, 431–441. 10.1083/jcb.141.2.431 9548721 PMC2148442

[B54] Zhen M. Jin Y. (1999). The liprin protein SYD-2 regulates the differentiation of presynaptic termini in *C. elegans*. Nature 401, 371–375. 10.1038/43886 10517634

[B55] Zinsmaier K. E. Eberle K. K. Buchner E. Walter N. Benzer S. (1994). Paralysis and early death in cysteine string protein mutants of *Drosophila*. Science 263, 977–980. 10.1126/science.8310297 8310297

[B56] Zinsmaier K. E. Hofbauer A. Heimbeck G. Pflugfelder G. O. Buchner S. Buchner E. (1990). A cysteine-string protein is expressed in retina and brain of *Drosophila*. J. Neurogenet. 7, 15–29. 10.3109/01677069009084150 2129171

